# Multiple variation patterns of terpene synthases in 26 maize genomes

**DOI:** 10.1186/s12864-023-09137-3

**Published:** 2023-01-27

**Authors:** Yang Sun, Wenqing Xiao, Qing-nan Wang, Jing Wang, Xiang-dong Kong, Wen-hui Ma, Si-xian Liu, Ping Ren, Li-na Xu, Yong-Jun Zhang

**Affiliations:** 1 Key Laboratory for Conservation and Use of Important Biological Resources of Anhui Province, Anhui Provincial Key Laboratory of Molecular Enzymology and Mechanism of Major Diseases, College of Life Sciences, Anhui Normal University, Wuhu, 241000 Anhui China; 2 State Key Laboratory for Biology of Plant Diseases and Insect Pests, Institute of Plant Protection, Chinese Academy of Agricultural Sciences, Beijing, 100193 China; 3 Institute of Plant Protection and Agro-products Safety, Anhui Academy of Agricultural Sciences, Hefei, 230031 China; 4 Institute of Bioinformatics, College of Agriculture and Biotechnology, Zhejiang University, Hangzhou, China

**Keywords:** Terpene synthases, Pangenome, Positive selection, Structural variation, Atypical, *Ostrinia furnacalis*

## Abstract

Terpenoids are important compounds associated with the pest and herbivore resistance mechanisms of plants; consequently, it is essential to identify and explore terpene synthase (*TPS*) genes in maize. In the present study, we identified 31 *TPS* genes based on a pan-genome of 26 high-quality maize genomes containing 20 core genes (present in all 26 lines), seven dispensable genes (present in 2 to 23 lines), three near-core genes (present in 24 to 25 lines), and one private gene (present in only 1 line). Evaluation of ka/ks values of *TPS* in 26 varieties revealed that *TPS25* was subjected to positive selection in some varieties. Six *ZmTPS* had ka/ks values less than 1, indicating that they were subjected to purifying selection. In 26 genomes, significant differences were observed in ZmTPS25 expression between genes affected by structural variation (SV) and those not affected by SV. In some varieties, SV altered the conserved structural domains resulting in a considerable number of atypical genes. The analysis of RNA-seq data of maize *Ostrinia furnacalis* feeding revealed 10 differentially expressed *ZmTPS*, 9 of which were core genes. However, many atypical genes for these responsive genes were identified in several genomes. These findings provide a novel resource for functional studies of *ZmTPS*.

**Supplementary Information:**

The online version contains supplementary material available at 10.1186/s12864-023-09137-3.

## Introduction

Terpenoids are a class of secondary metabolites produced by plants. Terpenoids are vital to photosynthesis, regulation of plant growth and development, pollination, and plant resistance to biotic and abiotic stressors [1]. For example, carotenoids such as tetraterpenoids can absorb and transmit light energy, and certain essential plant hormones (such as abscisic acid, oleuropein lactone, and gibberellin) are terpene derivatives that can influence plant growth and development. Linalool and nerolidol produced by tea under low temperatures can improve cold tolerance [2]. Linalool [3], limonene [4], nerolidol [5], and γ-terpinene [6] can inhibit the activity of rice leaf blight fungus. In addition, terpenoids are involved in induced defence responses in plants; for example, (E)-β-farnesene repels aphids [7, 8], while sphagnum and linalool attract rice louse tassel wasps [9].

Terpene synthase (TPS) catalyses the production of terpenoids, whose abundance is strongly linked to a large number of terpene synthase genes in plants [10]. With the exception of the moss *Physcomitrella patens*, which has only one functional *TPS* gene, the *TPS* gene family in plants is medium-sized. Other plant genomes contain approximately 20–152 *TPS* genes, although some have lost their functions over the course of their evolution [11].

Based on sequence characteristics, the *TPS* gene family is classified into three major classes and seven subfamilies, namely *TPS-a, −b, −c, −d, −e/f, −g*, and *-h.* Among them, genes in *TPS*-a are sesquiterpene synthases. *TPS-b* encodes monoterpene synthases exclusive to angiosperms, with a conserved R(R)X8W motif that functions in the initiation phase of the isomerisation cyclisation reaction or stabilises the protein through electrostatic interactions. The *TPS-g* subfamily is closely related to *TPS-b*, but its encoded protein lacks the conserved R(R)X8W motif and may produce acyclic, monocyclic, sesquiterpene, and diterpene products. *TPS-c* is found in terrestrial plants and is characterised by the ‘DxDD’ motif. *TPS-d* is gymnosperm-specific gene that encodes monoterpene, sesquiterpene, and diterpene synthases. The *TPS-e/f* subfamily is found mostly in vascular plants, encodes copalyl diphosphate synthase (CPS) and kaurene synthase (KS), and is responsible for gibberellin biosynthesis [10, 12, 13]. *TPS-h* is only present in the *selaginella tamariscina* which encode ‘DXDD’ and ‘DDXXD’ motifs [11].

The study of *ZmTPS* genes is crucial as terpenoids play vital roles in pest and herbivore resistance; however, the *TPS* gene family in maize has not yet been identified and analysed. Traditional gene family identification approaches generally involve a genome-wide search for conserved structural domains of the gene family, followed by the identification of the gene family members after confirmation of the conserved structural domains. However, a single reference genome-based approach to gene family identification cannot identify the gene family members that are missing from the reference genome but are present in other genomes. Hufford et al. published a maize pan-genome based on 26 high-quality genomes containing a large amount of presence-absence variation (PAV) and SV information, thus laying the foundation for gene family and functional studies [14].

The present study identified 31 *TPS* genes based on a pan-genome of 26 high-quality maize genomes, which included 20 core genes, seven dispensable genes, three near-core genes, and one private gene. The analysis of ka/ks values of *TPS* in 26 varieties revealed the effect of SV on gene structure, expression, and conserved structural domains. In some varieties, SVs impair conserved *ZmTPS* structural domains resulting in a significant number of atypical genes. Ten *ZmTPSs* showed differential expression when the RNA-seq data from *Ostrinia furnacalis-*infested maize samples were analysed. These results provide a novel resource for functional studies of *ZmTPS* genes.

## Materials and methods

### Identification of maize *TPS* gene family

The 26 maize genomes were obtained from a study by Hufford et al. [14]. The hidden Markov model (HMM) profiles of the *TPS* N-terminal domain (PF01397) and *TPS*-C-terminal domain (PF03936) were retrieved from the Pfam database (http://pfam.xfam.org/). *TPS* domains were searched using HMMER 3.3.2, with a threshold of e < 1E-5. The *TPS* candidates were submitted to SMART (http://smart.embl-heidelberg.de/) [15] to confirm the existence of *TPS* N and *TPS* C terminal domains. Finally, among the 26 maize accessions, genes with a collinear relationship to *TPS*s were considered *TPS* members.

### Phylogenetic analysis and presence/absence variation of *ZmTPS* gene family

The protein sequences of the *TPS*s from *Arabidopsis* and maize were used for phylogenetic analysis. Multiple sequence alignments were performed using MAFFT v7.490 [16], and a phylogenetic tree was constructed using FastTree version 2.1.11 [17]. The final graphics were generated using iTOL v6 (https://itol.embl.de/) [18].

The PAV information of *TPS*s was obtained from Hufford et al. [14]. Heatmap was used to depict the presence/absence of each *TPS* in the 26 accessions using Rscript version 4.0.3 and the ComplexHeatmap package [19].

### Ka/Ks calculation

The protein and coding sequence (CDS) sequences of *ZmTPS* genes in 26 maize genomes were obtained from the study by Hufford et al .[14]. *ZmTPS* sequences were compared, and Ka/Ks values were calculated using the KaKs Calculator [20]. The R packages ggridges and ggplot2 (v4.0.3) were used to create the Ridgeline plot of ka/ks values [21, 22]. The heatmap of the proportion of *ZmTPS* genes with Ka/Ks values greater than one was plotted using a ComplexHeatmap R (v4.0.3) [19].

### Analysis of the expression of *TPS*s overlapped with SVs

The location of SV in each variety and the gene expression data in 26 accessions were obtained from Hufford et al. [14]. An in-house Perl script was used to determine whether the *TPS*s overlapped with SVs in each variety. If the *TPS* overlapped with SVs, the *TPS* expression data in this variety were considered as the expression data of genes with SVs; otherwise, they were considered as the expression data of genes without SVs. The Pearson correlation coefficients were calculated between the presences of SVs overlapped with genes and gene expression level [23]. And *TPS*s with *p* < 0.05 and |r| > 0.3 were considered to have significantly altered expression levels due to SVs. Significant differences between atypical and typical *TPS* genes were determined by wilcox test.

### Analysis of the gene structure of *ZmTPS*s

Genome annotation files in general feature format (GFF) were downloaded from http://maize-pangenome.gramene.org. MEME Suite v5.4.1 (https://meme-suite.org/meme/tools/meme) was used to analyse the protein sequences of *ZmTPS,* whose expression levels were significantly altered by SV, with the number of motifs set to 10. Subsequently, TBtools (v1.098761) was used to map gene structure using the files generated in the preceding step and the gff file [24].

### Analysis of conserved structural domains of *TPS* under the influence of SV

The ZmTPS protein sequences of the most SV-overlapped genome (Ms71) and the reference genome (B73) were uploaded to MEME Suite v5.4.1 (https://meme-suite.org/meme/tools/meme), with the number of motifs set to 10, to obtain two weblogos of TPS protein sequences from the two genomes. The R package ComplexHeatmap was used to plot the presence of typical genes (containing completely conserved structural domains) and atypical genes (not containing completely conserved structural domains) for each *TPS* in the various maize genomes.

### RNA-seq data analysis

The expression value (reads per kilobase of transcript, per million mapped reads; RPKM) and results of the differential expression analysis of control and maize samples fed upon by *Ostrinia furnacalis* were retrieved from the supplementary tables in the study by Guo et al. [25]. And other RNA-seq data about maize treated with oral secretions (OS) of *Mythimna separata* were also download [26, 27]. Low quality sequences were remove by fastp [28]. The clean reads were mapped to the reference genome by hisat 2[29]. HTSeq was used to obtain the reads counts for each gene [30]. Differentially expressed genes were defined using a threshold of |log2FoldChange| > 1 and FDR ≤ 0.05. Heatmaps were generated with log2 (RPKM + 1) values using R (v4.0.3) and the ComplexHeatmap package (v2.6.2) [19].

## Result

### Pan-genome-wide *ZmTPS* identification

**Fig. 1:**
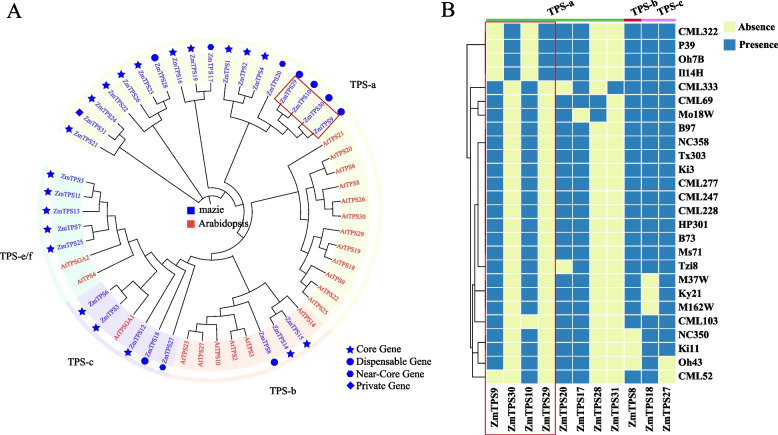
**A** Phylogenetic tree of maize and Arabidopsis *TPS* genes. **B** Heatmap of the presence and absence of 11 *TPS*s in 26 maize varieties except for the core genes. The genes in red boxes represent possible mutually exclusive PAV genes

In the present study, 32 *ZmTPS* genes were identified in the maize pan-genome, of which 20 were core genes, seven were dispensable genes, three were near-core genes, and one was a private gene. In the reference genome, 29 *TPS* were identified, three of which were atypical, and four *ZmTPSs* contained both typical and atypical genes. The supplementary table pan_gene_matrix_v3_cyverse.csv by Hufford et al. contained the sequence of the maize ZmTPS protein that was used to construct the evolutionary tree [14].. Based on the *OsTPS* categorisation information [31], we classified the *ZmTPS* (Fig. 1A) into four subgroups. *TPS-a* was the largest group, containing 18 *TPS* genes, followed by *TPS-c* and *TPS-e/f*, each containing five *TPS* genes. *TPS-b* was the smallest group, with only three *TPS*. All the genes in *TPS-e/f* were core genes. Figure 1B shows the presence or absence of *ZmTPS* genes other than the core genes in the 26 maize varieties. *TPS31* and *TPS28* were present in only one (CML333) and two (CML6 and Mo18W) varieties, respectively, suggesting that these two genes may be associated with traits specific to these varieties.

### *ZmTPS* is subjected to different selection pressures among maize varieties

**Fig. 2:**
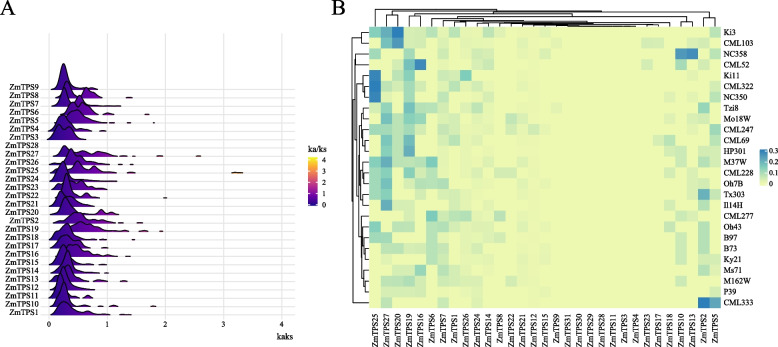
Ka/Ks values of *ZmTPS*. **A** Distribution of Ka/Ks values of *ZmTPS* in 26 maize varieties. **B** Heatmap of the frequency of occurrence of different maize varieties at each *TPS* with Ka/Ks ratio > 1

Analysis of ka/ks values can reveal the selection pressure on gene family members in different varieties forming. To explore the selection pressure on *ZmTPS* genes, we calculated the Ka/Ks value for each *TPS* gene based on the gene sequences in the 26 maize genomes (Fig. 2). Figure 2A depicts the Ka/Ks values of *TPS* in 26 maize cultivars. *ZmTPS28* was present in only two varieties and thus could only produce one Ka/Ks value, whereas *TPS31* was present in only one variety; thus, its Ka/Ks value could not be determined. All the Ka/Ks values of *TPS* genes peaked between 0 and 1, although the location of the peaks varied, with *ZmTPS11* having the highest Ka/Ks value. *TPS25* was positively selected for some varieties, as indicated by the Ka/Ks values, which ranged from 3 to 4. Six *ZmTPS* genes were found to have Ka/Ks values less than one, indicating that these genes were subjected to purifying selection. In the 26 maize genomes, *TPS25*, *TPS 27*, *TPS20*, *TPS19*, *TPS16*, *TPS2*, and *TPS5* had a large proportion of Ka/Ks values greater than one (Fig. 2B), indicating that these genes were under selection pressure during maize domestication.

### Expression and structure of *ZmTPS25* genes are affected by SV

**Fig. 3:**
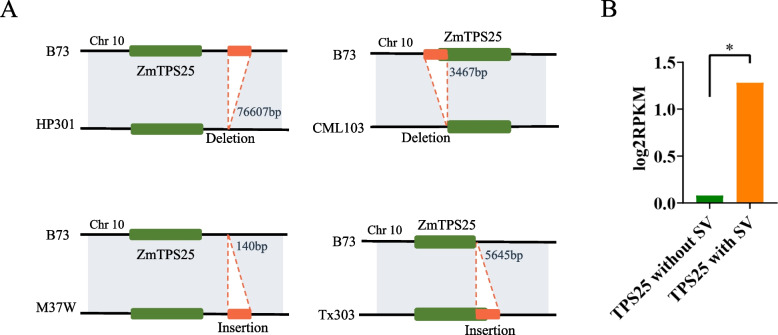
Effect of SV on genes. **A** The effects of SV insertion and deletion on *ZmTPS25*. **B** The expression of *TPS25* was significantly affected by SVs

A total of 478 SVs overlapped with 30 gene regions of *ZmTPS* as well as upstream and downstream 2-kb regions. Compared with the reference genome, SVs were characterised as deletions, insertions, inversions, and duplications (Fig. 3A). Pearson correlation coefficients were determined for the expression values of genes that overlapped with SV and genes that did not. The results revealed a significant difference between genes with and without SV only for *ZmTPS25* (*p* < 0.05 and |r| > 0.3), indicating that SV significantly altered *ZmTPS25* expression (Fig. 3B).

**Fig. 4:**
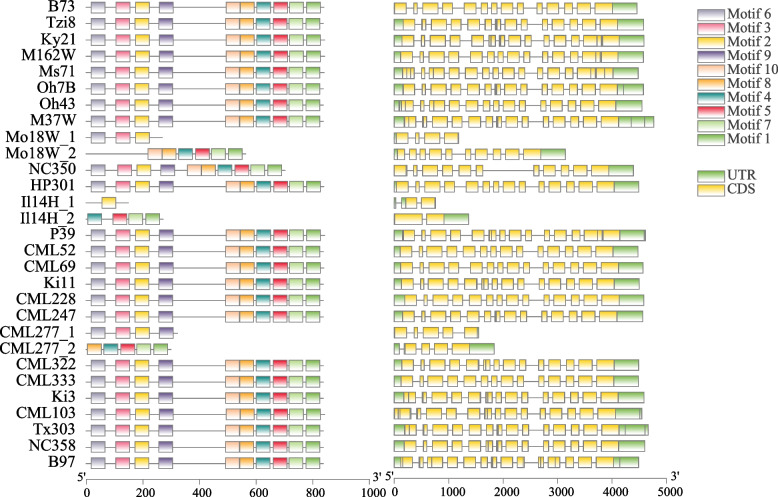
Gene structure of *ZmTPS25* in 26 maize genomes

We analysed the gene structure of *ZmTPS25* in 26 maize genomes to explore whether SV affected the *TPS25* gene structure (Fig. 4). The domains of *ZmTPS25* in most maize genomes were consistent with the reference genome (B73). However, *ZmTPS25* structural domains in Mo18W, I114H, and CML277 were significantly altered, while one *ZmTPS25* gene in I114H had only one exon.

### Atypical *ZmTPS* genes in the maize genomes

**Fig. 5:**
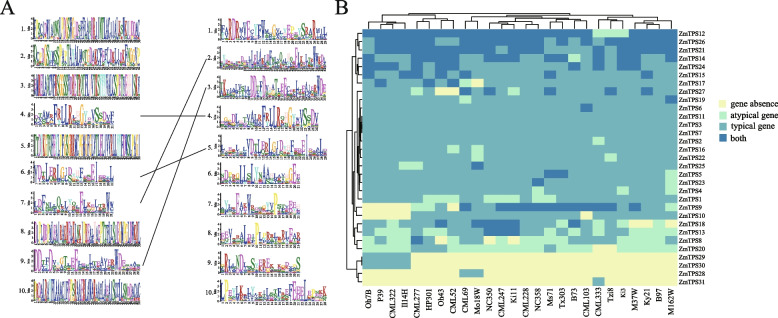
The conserved structural domains of *TPS* were affected by SVs. **A** The weblogos of the Ms71 *ZmTPS* and the reference genome are shown on the left and right, respectively. The weblogos connected by the lines indicate that they are corresponding. Weblogos are arranged in the order of E-value. **B** Heatmap showing the typical or atypical nature of each *TPS* gene in each variety. ‘both’ represents the presence of both typical and atypical genes

To explore the effects of SVs on the conserved structural domains of *ZmTPS* in different maize genomes, we selected Ms71, the variety with the highest number of *TPS* genes overlapping with SV, for conserved structural domain comparison with the reference (Fig. 5A). Four out of 10 motifs of *TPS* in Ms71 corresponded to the reference, but the remaining six did not. Furthermore, the amino acids in each set of mutually corresponding motifs did not perfectly match, indicating a strong effect of SV on the conserved structural domains of different *ZmTPS* genes. Because gene family identification is based on conserved structural domain searches, we calculated the number of typical (containing conserved structural domains) and atypical genes (without conserved structural domains) in each species (Fig. 5B). In most varieties, the atypical genes were *ZmTPS13*, *ZmTPS8*, and *ZmTPS20*. These findings imply that SVs influence *ZmTPS* conserved structural domains, leading to a significant number of atypical genes.

### Atypical TPS genes were widely expressed in maize

**Fig. 6:**
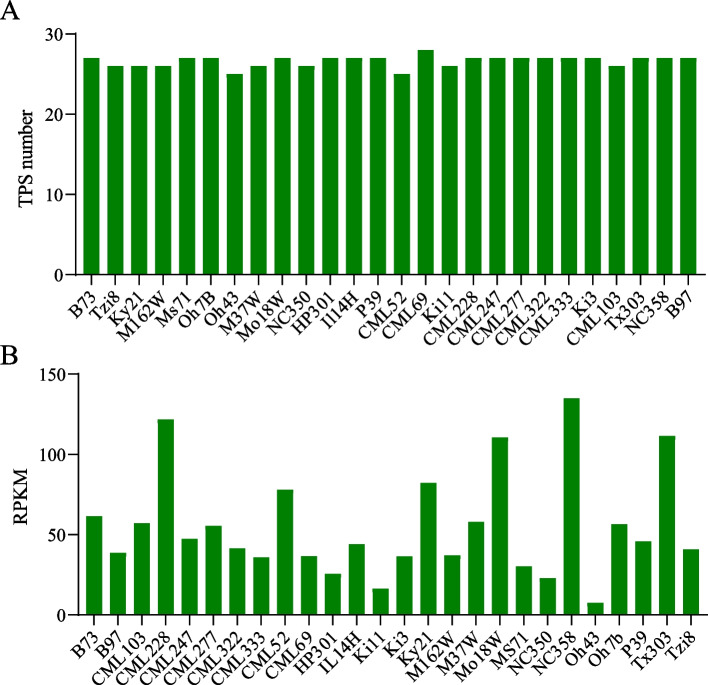
Number of *ZmTPS* (**A**) and total expressed dose (**B**) in 26 maize genomes

To explore whether there is a correlation between the number and total expression dose of *ZmTPS* among different varieties, we counted the number and total expression values of *ZmTPS* in each variety (Fig. 6). Each variety had almost the same number of *ZmTPS* genes, but the overall expression dose varied greatly, with a minimum total FPKM value of 7 (Oh43) and a maximum of 134.99 (NC358). In this study, we found that the absence of *ZmTPS* genes did not directly cause the total change in expression dose. For example, in CML52, the number of *TPS* genes was 25 and the total FPKM value was 77.88, whereas the number of *TPS* genes in CML69 was 28 and the total FPKM value was 36.72. In addition, some varieties had a small number of *TPS* genes that had low total *TPS* expression values; for example, there were only 25 *TPS* genes in Oh43, and the total FPKM value was 7.58. Pearson correlation analysis of the number of *TPS* and log2RPKM values for each species revealed that r = 0.31 and *p* = 0.12 (Fig. S2), indicating that the number of *ZmTPS* genes and the total expression dose were not correlated.

**Fig. 7:**
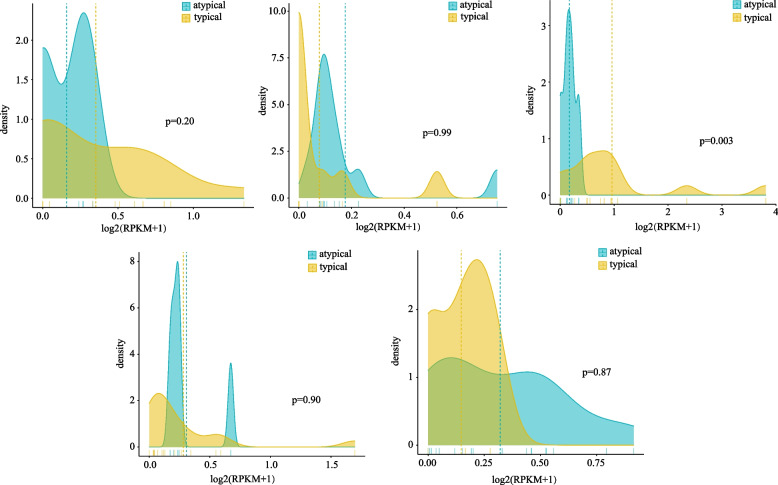
Expression density distribution of typical and atypical genes in 26 maize genomes. In order, *ZmTPS1*, *ZmTPS8*, *ZmTPS13*, *ZmTPS18* and *ZmTPS20*

In 26 maize genomes, *ZmTPS1*, *ZmTPS8*, *ZmTPS13*, *ZmTPS18,* and *ZmTPS*2 had more than three atypical genes. Expression analysis showed that the expression of typical and atypical genes had different distribution patterns (Fig. 7). For example, the expression value distribution of *ZmTPS13* was significantly higher in maize varieties with typical structures than those with atypical structures. Atypical genes were widely expressed and distributed with high levels of expression in *ZmTPS8* and *ZmTPS20*, but this difference was not statistically significant.

### The response of *ZmTPS* to the *Ostrinia furnacalis* feeding

**Fig. 8:**
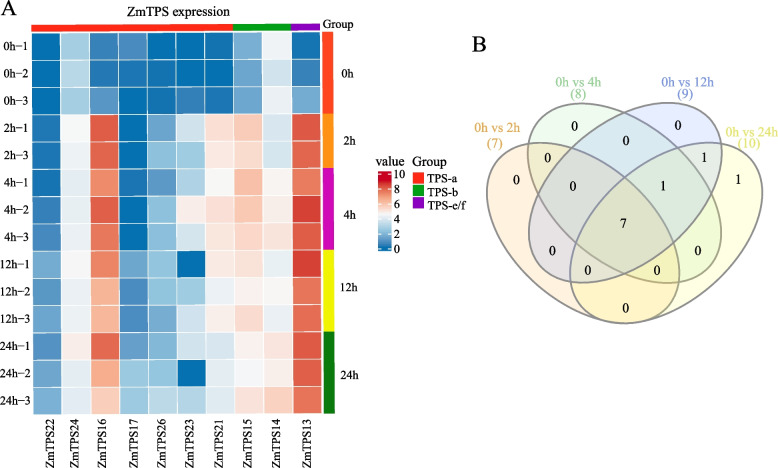
Response of *ZmTPS* to *Ostrinia furnacalis* feeding. **A** Heatmap of differentially expressed *ZmTPS* expression in different samples. The value in the graph was log2(FPKM+ 1) value. **B** Venn Diagrams of overlapping and specific differentially expressed *ZmTPS* genes for different comparisons

The Asian corn borer (*Ostrinia furnacalis* Guenée) is an agricultural pest of several crops, mainly corn [32]. To analyse the response of *ZmTPS* genes to Asian corn borer feeding, we obtained differential expression data at different time points following Asian corn borer feeding from the study of Guo et al. [25]. All differentially expressed *ZmTPS* were upregulated in response to Asian corn borer feeding. Among all differentially expressed *ZmTPS*, *ZmTPS13* and *ZmTPS16* had higher expression levels after corn borer feeding (Fig. 8A). The number of differentially expressed *TPS* increased with feeding time, reaching ten after 24 h, accounting for 31.25% of the total number of *ZmTPS*, indicating that the *ZmTPS* gene family plays a vital role in response to corn borer infestation (Fig. 8B).

## Discussion

The maize pan-genome consists of 26 high-quality genomes containing the genes absent in the reference genome. Compared to reference genome-based gene family analysis, pan-genome-based analysis is more comprehensive and can identify non-reference *ZmTPS* genes. We identified three non-reference *TPS* genes and 29 *TPS* genes in the reference genome based on the maize pan-genome. More importantly, even if a gene is present in the reference genome, it might not be present in other maize genomes. Only 20 of the 32 *ZmTPS* genes were present in all the varieties. This phenomenon was observed in different species. For example, a recent pan-genomic *TPS* gene analysis in rice found that one *OsTPS* gene was missing from the rice reference genome [33]. However, although the *ZmTPS* numbers differed, the varieties with fewer *ZmTPS* genes did not show low total *TPS* gene expression. This could be due to many reasons, including the fact that some gene family members have similar functions and can compensate for one another. Consequently, when one or more genes are absent, expression of the spare genes increases to preserve the robustness of plant life activities. Some investigations have identified homologous genes with mutually compensatory mechanisms, such as the ability of paralogous *CLV1* homologues to be upregulated in the absence of *CLV1* gene activity, thus acting as a compensatory mechanism to replace *CLV1* in stem cell endocytosis [34]. *ZmTPS10*, *ZmTPS9*, *ZmTPS29*, and *ZmTPS30* were closely related in the phylogenetic tree, and none of them were core genes; however, the PAV heatmap (Fig. 1B) shows that none of them are simultaneously missing in any of the varieties. The mutually exclusive absence suggests that these four genes are functionally redundant with each other but will not be absent simultaneously to ensure the functioning of these genes.

In the present study, SV was found to affect the expression of *ZmTPS25*, which warrants further investigation. Previous studies showed that the promoter region of maize bZIP68 differs from the ancestral species *Z. mays ssp. parviglumis* and *Z. mays ssp. mexicana*. A 358-bp fragment insertion at the − 972 (B73) locus upregulated the expression of bZIP68 in maize, leading to a reduction in cold tolerance [35, 36]. The effect of SV on the promoter or downstream regions of the *ZmTPS25* gene is shown in Fig. 3A, suggesting that structural variation in gene regions can also affect gene expression, as reported in a tomato pan-SV study [37]. SVs affected both *ZmTPS* expression as well as the gene sequence and structure. This may result in the absence of some domains, which would prevent the identification of gene family members using conventional bioinformatics techniques. However, gene family members lacking these regions may still have vital biological functions. For example, Zhao et al. identified a *Ptr* gene on rice chromosome 12 that encoded an unusual broad-spectrum disease resistance protein and contained a two-bp deletion resulting in a truncation of the encoded protein [38]. While maize pan-genomes were constructed based on multiple high-quality genomes, atypical gene family members arising due to SV affecting multiple genomes can be detected by a collinear relationship between genomes. SVs may also alter the number of gene exons. For example, *ZmTPS25* showed variations in the number of exons in different varieties. These genes with a reduced number of exons may belong to atypical gene families but are still functional. For example, Zheng et al. identified an atypical *LEA* gene in *Ipomoea pes-caprae* L. with only one exon that confers salt/drought and oxidative stress tolerance [39]. Therefore, this study identified several atypical *TPS* genes in maize, providing additional resources that can aid in better understanding the function of *TPS* in maize.

A total of 10 *TPS* genes were differentially expressed in maize in response to *Ostrinia furnacalis* feeding, of which only *ZmTPS17* was a near-core gene, and the others were core genes. This suggests that *ZmTPS*, which is conserved in maize with minimal gene absences, may be implicated in the response of plants to insect feeding. *ZmTPS17*, a gene encoding monoterpene synthase, has been found to be upregulated in other studies on insect feeding on maize [40], indicating that it may be an important insect feeding response gene. *ZmTPS14*, a gene of the *TPS-b* class involved in the synthesis of sesquiterpenoids but not in the same subgroup as *ZmTPS17*, was induced after feeding by the Asian corn borer *Ostrinia furnacalis* and the larvae of the herbivorous beetle *Holotrichia parallela* [41]. *TPS15*, a gene adjacent to *TPS14* in the phylogenetic tree, catalyses the production of linalool, (E)-nerolidol, and (E, E)-geranyllinalool, the expression of which has been shown to be induced by mechanical damage to maize seedlings combined with the oral secretion of caterpillars [42], suggesting that *TPS15* is also involved. These results indicate that *TPS* genes are critical for insect resistance in maize and are entirely conserved, except for *ZmTPS17*, which was absent in Mo18W. However, we found that some varieties have atypical genes, such as the novel *TPS14* copies in the genomes of M162W, M37W, and Ki3, which have both typical and atypical genes. These atypical *ZmTPS* genes warrant further investigation.

### Supplementary Information

**Additional file 1: Fig. S1.** The ratio of SV to typical and atypical genes overlap. **Fig. S2.** Scatter plot of *ZmTPS* gene number and total expressed dose correlation analysis. **Fig. S3.** The differentially expressed *ZmTPS* genes in other studies. **Table S1.** The ZmTPS names and their corresponding gene names in multiple maize genomes. **Table S2.** The atypical ZmTPS genes in maize genomes.

## Data Availability

The RNA-seq data were obtained from the NCBI SRA database (PRJNA531363) and Genome Sequence Archive of BIG Data Center (PRJCA006850). The maize genomes were downloaded from maize pangenome database (http://maize-pangenome.gramene.org). Data supporting the findings of this work are available within the paper and its Supplementary Information files. The datasets generated and analyzed during the current study are available from the corresponding author upon request.
