# Supplementary material for: Multiple variation patterns of terpene synthases in 26 maize genomes
Source: BMC Genomics. 2023 Jan 27;24:46. doi: 10.1186/s12864-023-09137-3 (PMC9881264; doi:10.1186/s12864-023-09137-3)
Supplement: Supplementary file 1 — Additional file 1: Fig. S1. The ratio of SV to typical and atypical genes overlap. Fig. S2. Scatter plot of ZmTPS gene number and total expressed dose correlation analysis. Fig. S3. The differentially expressed ZmTPS genes in other studies. Table S1. The ZmTPS names and their corresponding gene names in multiple maize genomes. Table S2. The atypical ZmTPS genes in maize genomes. [file 12864_2023_9137_MOESM1_ESM.zip › Attachment description_revised_version.docx]

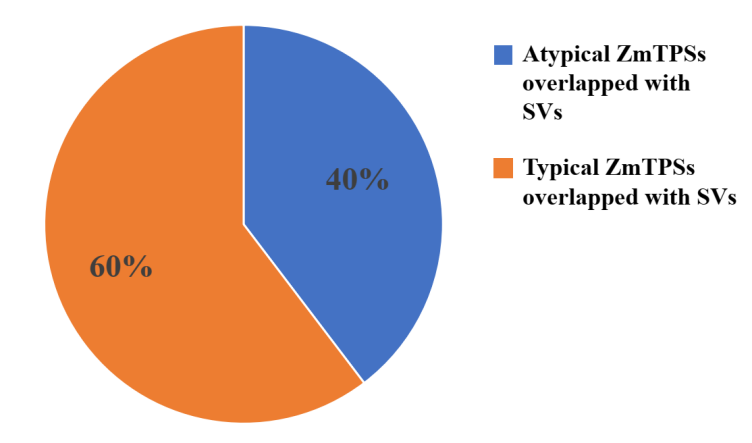


Figure S1 The ratio of SV to typical and atypical genes overlap.


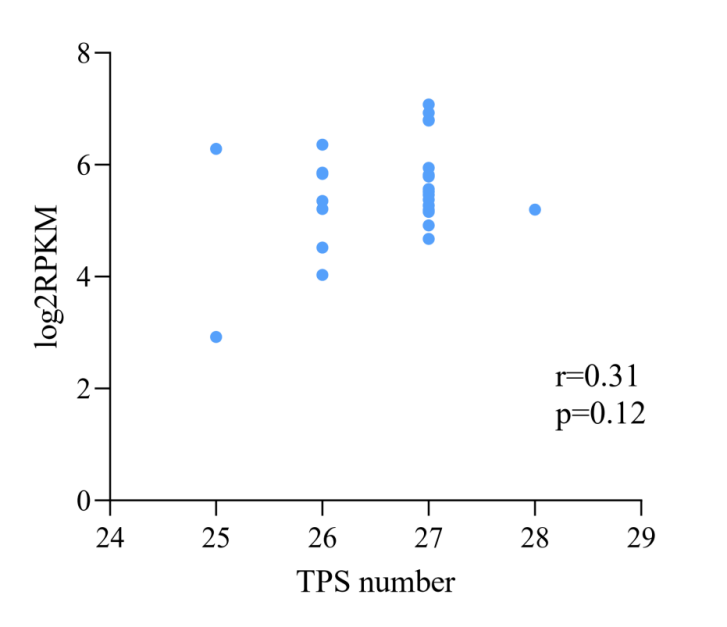


Figure S2 Scatter plot of *ZmTPS* gene number and total expressed dose correlation analysis.


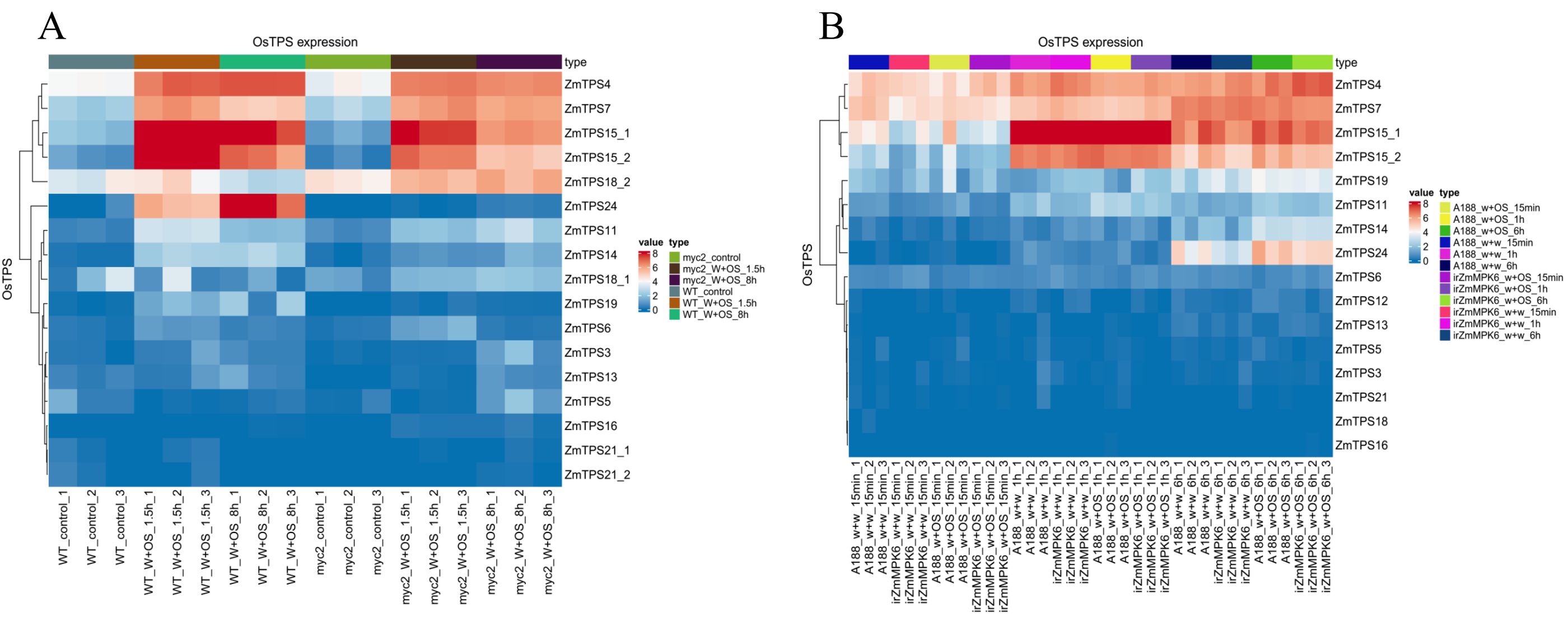


Figure S3 The differentially expressed *ZmTPS* genes in other studies. A, The study of JA-mediated response process of insect resistance defense. The sample groups are: myc2: myc2 mutant lines; control: mock treated samples; W+W: treated with water; W+OS: applying the oral secretions (OS) of *Mythimna separata* to the wounded surface. B, Simulation of insect feeding study. The sample groups are: A188: maize inbred line A188; irZmMPK6：ZmMPK6-silenced maize; w+w: the wounds were treated with 20 μl of water; w+OS: the wounds were treated with *Mythimna separata* OS.

Table S1: The ZmTPS names and their corresponding gene names in multiple maize genomes.

Table S2: The atypical ZmTPS genes in maize genomes.
