# Supplementary figures and images for: Multiple variation patterns of terpene synthases in 26 maize genomes
Source: BMC Genomics. 2023 Jan 27;24:46. doi: 10.1186/s12864-023-09137-3 (PMC9881264; doi:10.1186/s12864-023-09137-3)

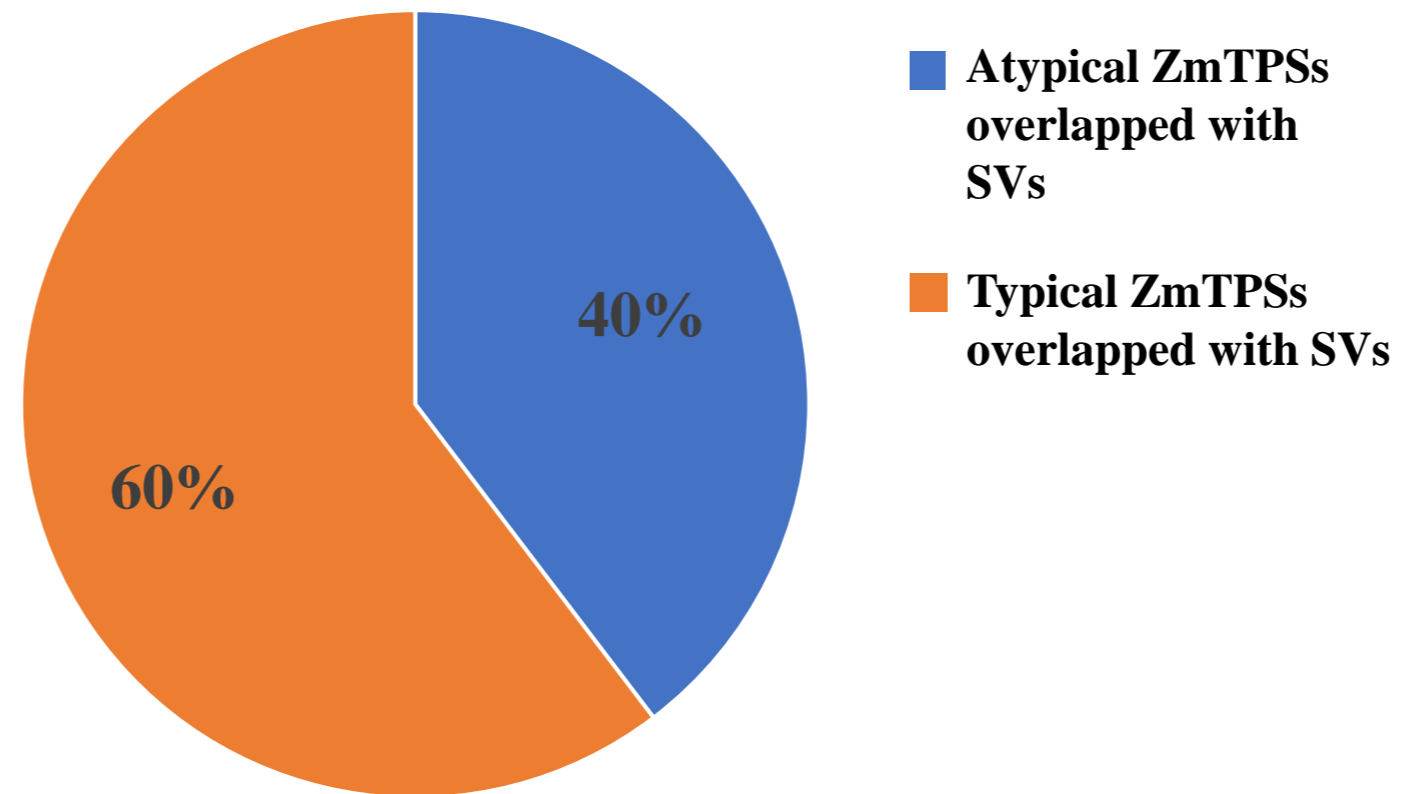

Supplement: Supplementary file 1 — Additional file 1: Fig. S1. The ratio of SV to typical and atypical genes overlap. Fig. S2. Scatter plot of ZmTPS gene number and total expressed dose correlation analysis. Fig. S3. The differentially expressed ZmTPS genes in other studies. Table S1. The ZmTPS names and their corresponding gene names in multiple maize genomes. Table S2. The atypical ZmTPS genes in maize genomes. [file 12864_2023_9137_MOESM1_ESM.zip › FigureS1.pdf]

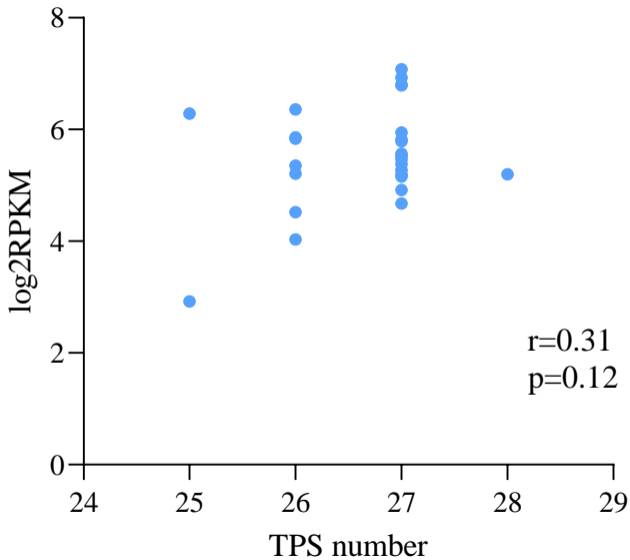

Supplement: Supplementary file 1 — Additional file 1: Fig. S1. The ratio of SV to typical and atypical genes overlap. Fig. S2. Scatter plot of ZmTPS gene number and total expressed dose correlation analysis. Fig. S3. The differentially expressed ZmTPS genes in other studies. Table S1. The ZmTPS names and their corresponding gene names in multiple maize genomes. Table S2. The atypical ZmTPS genes in maize genomes. [file 12864_2023_9137_MOESM1_ESM.zip › FigureS2.pdf]

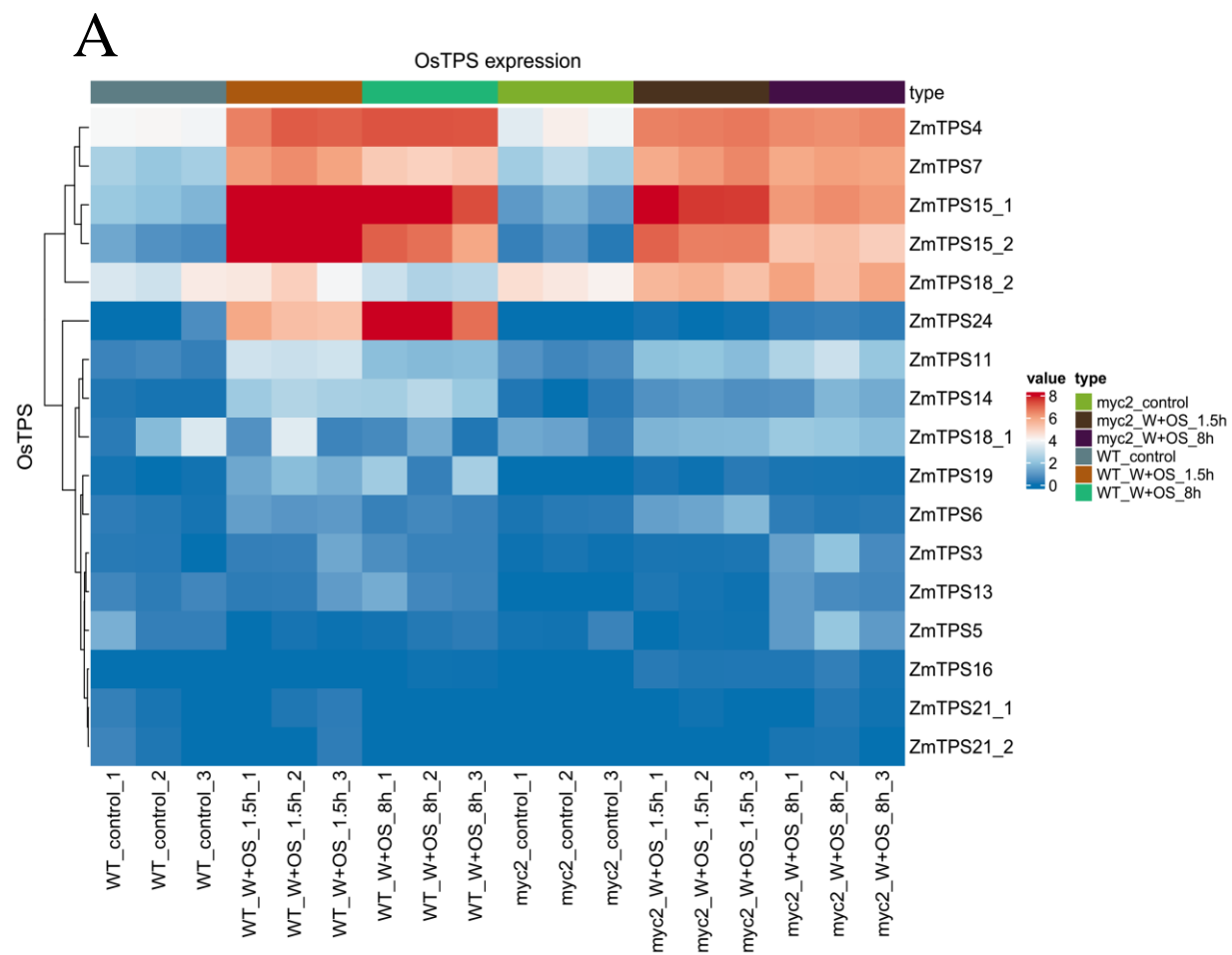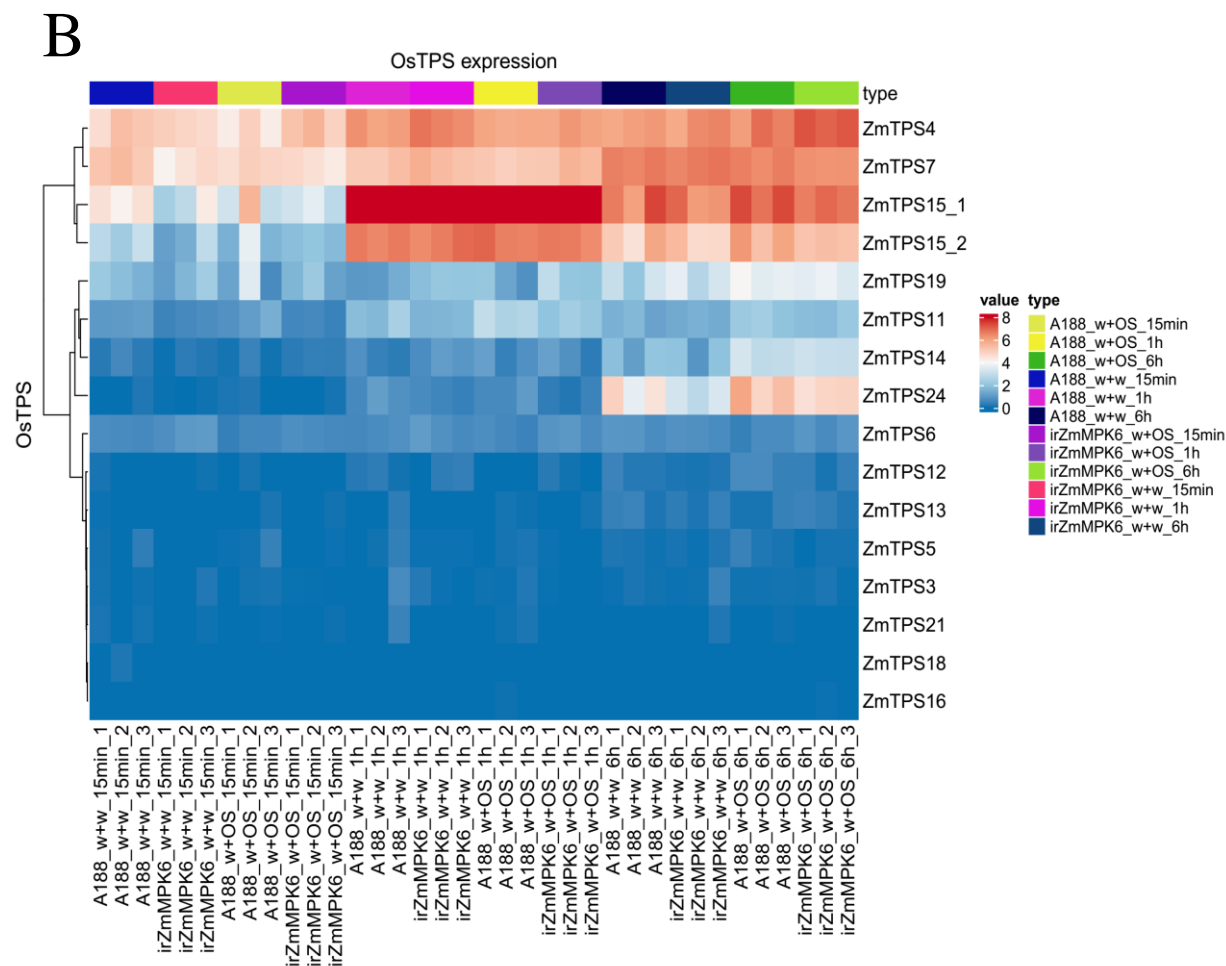

Supplement: Supplementary file 1 — Additional file 1: Fig. S1. The ratio of SV to typical and atypical genes overlap. Fig. S2. Scatter plot of ZmTPS gene number and total expressed dose correlation analysis. Fig. S3. The differentially expressed ZmTPS genes in other studies. Table S1. The ZmTPS names and their corresponding gene names in multiple maize genomes. Table S2. The atypical ZmTPS genes in maize genomes. [file 12864_2023_9137_MOESM1_ESM.zip › FigureS3.pdf]
